# Short and Long-Term Innovations on Dietary Behavior Assessment and Coaching: Present Efforts and Vision of the Pride and Prejudice Consortium

**DOI:** 10.3390/ijerph18157877

**Published:** 2021-07-25

**Authors:** Desiree A. Lucassen, Marlou P. Lasschuijt, Guido Camps, Ellen J. Van Loo, Arnout R. H. Fischer, Roelof A. J. de Vries, Juliet A. M. Haarman, Monique Simons, Emely de Vet, Marina Bos-de Vos, Sibo Pan, Xipei Ren, Kees de Graaf, Yuan Lu, Edith J. M. Feskens, Elske M. Brouwer-Brolsma

**Affiliations:** 1 Division of Human Nutrition and Health, Wageningen University & Research, Stippeneng 4, 6708 WE Wageningen, The Netherlands; desiree.lucassen@wur.nl (D.A.L.); marlou.lasschuijt@wur.nl (M.P.L.); guido.camps@wur.nl (G.C.); kees.degraaf@wur.nl (K.d.G.); edith.feskens@wur.nl (E.J.M.F.); 2 Marketing and Consumer Behavior Group, Wageningen University & Research, Hollandseweg 1, 6706 KN Wageningen, The Netherlands; ellen.vanloo@wur.nl (E.J.V.L.); arnout.fischer@wur.nl (A.R.H.F.); 3 Biomedical Signals and Systems, University of Twente, Drienerlolaan 5, 7522 NB Enschede, The Netherlands; r.a.j.devries@utwente.nl; 4 Human Media Interaction, University of Twente, Drienerlolaan 5, 7522 NB Enschede, The Netherlands; j.a.m.haarman@utwente.nl; 5 Consumption and Healthy Lifestyles, Wageningen University & Research, Hollandseweg 1, 6706 KN Wageningen, The Netherlands; monique.simons@wur.nl (M.S.); emely.devet@wur.nl (E.d.V.); 6 Faculty of Industrial Design Engineering, Delft University of Technology, Landbergstraat 15, 2628 CE Delft, The Netherlands; M.Bos-DeVos@tudelft.nl; 7 Systemic Change Group, Department of Industrial Design, Eindhoven University of Technology, Atlas 7.106, 5612 AP Eindhoven, The Netherlands; s.pan1@tue.nl (S.P.); x.ren@tue.nl (X.R.); y.lu@tue.nl (Y.L.); 8 School of Design and Arts, Beijing Institute of Technology, 5 Zhongguancun St. Haidian District, Beijing 100081, China

**Keywords:** technological innovations, dietary assessment, behavior change interventions, coaching, development, apps, images, conversational agent, artificial intelligence, machine learning

## Abstract

Overweight, obesity and cardiometabolic diseases are major global health concerns. Lifestyle factors, including diet, have been acknowledged to play a key role in the solution of these health risks. However, as shown by numerous studies, and in clinical practice, it is extremely challenging to quantify dietary behaviors as well as influencing them via dietary interventions. As shown by the limited success of ‘one-size-fits-all’ nutritional campaigns catered to an entire population or subpopulation, the need for more personalized coaching approaches is evident. New technology-based innovations provide opportunities to further improve the accuracy of dietary assessment and develop approaches to coach individuals towards healthier dietary behaviors. Pride & Prejudice (P&P) is a unique multi-disciplinary consortium consisting of researchers in life, nutrition, ICT, design, behavioral and social sciences from all four Dutch Universities of Technology. P&P focuses on the development and integration of innovative technological techniques such as artificial intelligence (AI), machine learning, conversational agents, behavior change theory and personalized coaching to improve current practices and establish lasting dietary behavior change.

## 1. Introduction

Globally, poor diet quality is acknowledged to significantly impact health, and associated health care costs. The Global Burden of Disease study even indicated that a suboptimal diet is the second-leading risk factor for disability-adjusted life years and deaths worldwide after tobacco [1].

Despite the importance of a healthy diet, nutrition guidelines set by the Dutch Health Council are often not met by Dutch consumers. To illustrate, the Dutch Health Council (2015) recommends daily intakes of ≥250 g of vegetables and ≥200 g of fruits, one portion of fatty fish per week (17 g/day) and to limit intake of red meat and processed meat (<43 g/day) [2]. According to the Dutch Food Consumption Survey 2012–2016, a substantial proportion of Dutch women and men (19–50 year) do not adhere to these recommendations, i.e., shown by self-reported mean daily intakes of 128 g and 131 g of vegetables, 115 g and 97 g of fruits, 14 g and 14 g of fish, along with 88 g and 131 g of meat (products), respectively [3]. Meeting healthy diet requirements is implicated in benefiting long-term health, i.e., a lower risk of overweight, obesity, coronary heart disease, stroke, diabetes, and colon and lung cancer [2]. Moreover, in order to prevent overweight and obesity, which often precede the above listed adverse health outcomes, food overconsumption (i.e., energy intake exceeding energy expenditure) should also be prevented [4]. In 2019, more than 50% of Dutch adults were overweight (volksgezondheidenzorg.info, accessed on 26 September 2020). This high overweight prevalence may to some extent relate to an obesogenic environment offering many high-caloric fast foods such as sugar-sweetened beverages and cakes/cookies [4,5,6,7,8,9,10,11,12,13]; often liquid foods or foods with a soft texture can be consumed quickly and in larger quantities compared to foods that require chewing [8,14,15]. It has for instance been shown that participants consuming a liquid product consumed 30% more of the product compared to participants consuming a semi-solid product despite similar palatability, energy density and macronutrient composition [16]. Therefore, targeting eating rate may reduce overconsumption and consequently excessive weight gain [17], for instance by stimulating the intake of hard textured foods such as a fruit salad instead of smoothies or by creating awareness of individual eating speed to stimulate people to eat more slowly.

To date, already many interventions have been designed to steer individuals to a healthier diet. However due to their ‘one-size-fits-all’ approach with merely generic advice catered to a (sub)population, these nutritional interventions have shown only limited success. To effectively influence dietary behaviors, interventions need to be more tailored to the individual’s needs and preferences [18,19], which is where mobile applications or technologies offer opportunities to coach individuals towards a healthier diet and prevent overconsumption [20]. However, at least three central questions need to be addressed. First, how to capture problems related to diet and eating behavior on an individual level? It is an enormous challenge to accurately quantify an individual’s food intake (i.e., food identification and portion size estimation) and as such to target personal diet optimization. There are many apps on the market that assess food intake but only very few are validated [21,22]. Validated dietary assessment apps are key to ensure the collection of reliable food intake data. Nevertheless, due to their self-report nature, even validated apps still contain various sources of error, such as inaccurate portion size estimates, inaccurate food identification, and incomplete recordings [23]. Second; how to tailor the dietary intervention to the individual? The adoption of behavior change theories is important to identify and influence key constructs related to behavior change. However, multiple reviews indicate that the integration of behavior change theories in diet-related apps is still limited [24,25,26]. Third, how to engage individuals in long-term use of diet-related apps? Perceived usability, perceived benefit, and trust in an app are important aspects for long-term user acceptance [27]. Moreover, intrinsic motivation is essential to commit to long-term use [28,29]. As existing diet-related apps are quite burdensome when related to food recording, decreasing this burden is assumed to beneficially impact user engagement, preferably while simultaneously improving the accuracy of the records. The above aspects can already be addressed in the developmental process by involving potential end-users as well as other stakeholders (intermediate users) (e.g., general practitioners, dieticians) [30], which can be especially valuable when developing for specific target groups (e.g., children, low SES, older adults). This approach is not only assumed to benefit app quality, but also trustworthiness of the app, the likelihood of professionals’ implementing the app in daily practice, and the prospect of adoption of the app by the target group [31].

New innovative technologies offer the opportunity for more personalized dietary interventions through apps, even more so by utilizing technologies such as wearables, different types of cameras, artificial intelligence (AI), and machine learning techniques [32,33,34]. The use of such smartphone applications allows for real-time personalized nutritional coaching, which requires less effort and has the potential to be more accurate compared to traditional methods [35]. However, the development of effective diet-related apps and technologies requires a multidisciplinary approach. Nutrition-, behavior change-, (AI) technology-, health- and human-technology interaction expertise is crucial to develop and implement dietary interventions that have an actual impact on health.

## 2. The Pride & Prejudice Consortium

**Figure 1:**
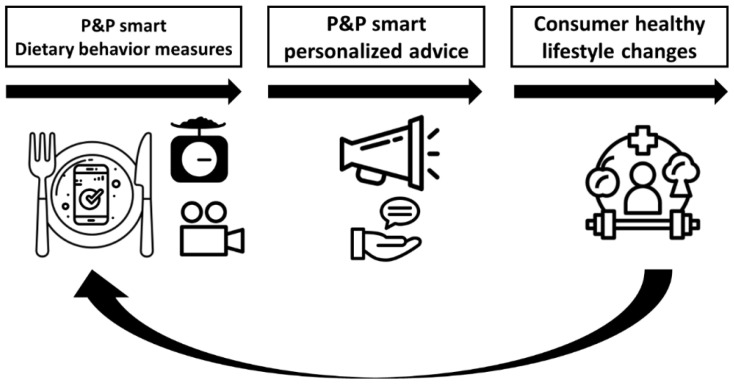
Overview of P&P efforts on technology driven dietary behavior assessment, and personalized interventions and coaching.

Pride & Prejudice (P&P) is a consortium that features a unique combination of disciplines, involving life, nutritional, ICT, design, behavioral and social sciences, and is composed of researchers from all four Dutch Universities of Technology (Delft University of Technology, Eindhoven University of Technology, University of Twente, and Wageningen University & Research). Within this consortium in-depth nutrition and nutrition behavior knowledge is combined with the technological knowledge of machine learning, lifestyle sensors, and knowledge of the design of effective health behavior change interventions. As such, P&P offers an exceptional platform to jointly develop new technology-based opportunities to build upon existing tools (Figure 1), for example by studying features that simplify the reporting of dietary intake as well as increase its accuracy by combining photo, video and/or speech recording with machine-learning or AI. This will not only facilitate a more accurate dietary behavior assessment among healthy adults, but may also aid assessment among specific target groups such as children and older adults. Moreover, the accuracy of dietary behavior assessment directly influences the efficacy of nutritional behavior coaching as dietary problems can be targeted more specifically [35]. Therefore, once a more accurate assessment can be achieved, individual, personalized dietary advice can be generated rather than general nutritional guidelines. P&P started its funded activities in January 2019 and will continue until January 2023. In this paper we outline our vision with respect to innovating our current dietary behavior assessment and intervention tools.

## 3. Current Tools to Assess Dietary Intake

Academia has long been investing considerable effort into creating validated tools to accurately assess dietary intake [21,23]. The mainstay of dietary assessment is based on self-report methods, i.e., food frequency questionnaires (FFQ), 24-h recalls (24hR), and food records. Originally, these methods were paper-pencil based but these evolved into computer- and web-based tools. The level of automatization within these tools has multiple benefits compared to traditional methods, e.g., decreased level of error, improved level of accuracy, increased user-friendliness, lower burden, and reduced costs. More recently, these dietary assessments tools were further innovated through the implementation of smartphone technology, enabled by almost universal adoption of smartphones in the population. Smartphone-based tools (i.e., apps) have the major advantage of enabling real-time data collection at any location at which the owner of the phone is present. Moreover, smartphones have multiple build-in sensors that may provide valuable information without any effort to provide input by the consumer (e.g., GPS, pedometer), and smartphones also offer the opportunity to easily connect to other apps/sensors (e.g., heart rate monitors, wearables). Eldridge et al. [21] created a clear overview of the available dietary assessment tools. Strikingly, whereas web-based tools are generally based on the FFQ or 24hR, all smartphone-based tools are based on the food record method.

The division of Human Nutrition and Health of Wageningen University & Research (WUR) created a solid foundation to assess diet in the general population by means of FFQs, 24hRs and food records [23]. Since the early 2000s, the original paper-pencil methods evolved into web-based tools (i.e., FFQ-tool™ and Compl-eat™) [23]. In addition, the most recent innovative tool developed by WUR researchers is the app “Traqq”, which can be used as a recall and food record. In the food record module, users can enter consumed foods throughout the day. Conversely, the recall module invites the user to record food intake in a specific time period by means of notifications. Once the user opens the app, access is given to an extensive food list based on the Dutch food composition database. The food list is flexible and can be modified by the researcher to fit different research purposes or different target groups (i.e., include sports nutrition or infant foods). Once a food item is selected, a consumed amount needs to be inserted, which can be reported in household measures (e.g., cups, spoons, glasses), standard portion sizes (e.g., small, medium, large), and weight in grams. The app also allows the user to enter all ingredients of an original recipe in combination with the quantity of the meal consumed. Yield and retention factors (i.e., retained weight and nutrients after cooking) are automatically taken into account. Traqq also enables linkage to online survey tools (e.g., Qualtrics). This way, additional questions can be incorporated linked to specific foods, eating occasions or times (e.g., context-related questions, mood questions) [36].

User-friendliness and trustworthiness of Traqq was addressed by consulting intended end-users during the development process, which eventually resulted in a clear and simple tool that can be used with minimal instruction. The effectiveness of this process was confirmed by the results of the evaluation study showing system usability scores (SUS) of 79/100, representing above average (SUS > 68) to excellent usability (SUS > 80) [36]. The logo of WUR, a respected research university, was incorporated in the app to underline solid scientific underpinning and enhance system credibility [37]. Currently, the validation of the recall-app is ongoing. Participants record their dietary intake by means of the app as well as validated traditional web-based dietary-assessment tools, with further validation using blood and urine samples. To evaluate upgrades since the previous evaluation, usability is reassessed as well. The preliminary results in terms of user-friendliness, validity and reproducibility seem promising; the final results are expected in mid-2021.

Traqq distinguishes itself from other dietary assessment apps in terms of its flexible nature. The app can be tailored to fit different research purposes in terms of the food list, portion size options, eating occasion or time questions, including additional questions and sampling scheme options. Moreover, it is possible to alternate between the recall and the food record module. To the best of our knowledge, Traqq is the only dietary assessment app that can also function as a recall, while other dietary assessment apps are all based on the food record method [21,36] that are prone to reactivity bias and social desirability bias, thus affecting dietary assessment accuracy [38,39]. In turn this will influence reliability of provided personalized advice and negatively influence the intervention’s efficacy.

In addition to self-report methods, there are also sensor-based technologies available that may facilitate dietary intake assessment (e.g., body-worn cameras, tooth sensors, smart dishes with weighing scales). Vu et al. [34] created an extensive overview of wearable dietary assessment technologies that aid objective food intake measurement, and as such eliminate various self-report related errors (e.g., estimation errors). These technologies are often invasive, not fully automated, or only provide partial food intake assessment; for example, only consumed amounts without food identification (smart dishes) or only a limited number of nutrients (tooth sensors). Consequently, these technologies are mostly exclusively used in a laboratory-setting and not validated for use in a real-life setting [33,34].

## 4. Current tools to Assess Eating Behavior

### 4.1. Individual Eating Behavior

Sensory-based technology such as weighing scales and video recordings can be used to assess dietary behavior (i.e., what and how much is consumed) and also to assess eating behavior (i.e., how is it consumed, e.g., chewing rate, bite size) [40,41]. Current gold standards to measure individual eating behavior are labor intensive and not suited for use beyond a controlled environment. To detect automated eating behaviors or oral processing such as chews and bites, participants are filmed while eating. Subsequently, recordings are annotated by two independent trained observers. Programs often used to annotate videos are Observer^®^ XT (Noldus Information Technology) and ELAN (Max Planck Institute) [42,43]. Chews and bites are annotated over time with readouts such as meal duration (i.e., time between first and last bite), eating rate (i.e., gram per minute), bite size (i.e., gram per bite) and oral processing duration (i.e., time between first chew and main swallow) [15,44]. Eating behavior and food texture (liquid, solid) can also be assessed with ear sensors that measure sound, PPG and accelerometry [41,45]. Oral processing in turn can also be measured with tracking dots (i.e., stickers on nose and chin) using Kinovea [46,47], which provides detailed and objective information on chew cycle duration, number of chews, and oral processing duration. Moreover, oral processing can be assessed by electromyography (EMG) or electromagnetic articulography (EMA), which tracks electrical activity of the jaw muscles [48,49]. EMG provides information on muscle effort or eating effort, number of chews, and chewing duration. EMA provides information on tongue movements, displacement, speed, and acceleration, which requires adhesive sensors on the tongue, jaw and cheeks. Other, more invasive methods of oral processing are video fluorograph and magnetic resonance imaging (MRI) that can be used to visualize the inside of the mouth and throat to track food and to observe movement of the jaw, tongue and esophagus [50,51]. The later methods are particularly suited to identify anatomical abnormalities and tracking of the swallowing process (e.g., evaluation of choking hazards in patients). To measure food properties in relation to eating behavior, bolus properties can be measured such as dry matter, particle size and mechanical properties [52]. Additionally, actual food intake (i.e., consumed amounts) is mostly measured by weighing before and after the meal.

As stated, most methods used to study eating behavior are not suited for field studies, but do allow for measures of food intake and eating behavior outside the lab [40,41]. Recently, WUR combined these technologies in a weighing-tray, hereafter referred to as the mEETr (derived from the Dutch words for ‘measurement device’ and ‘to eat’). The mEETr consists of a regular dining tray with three built-in weighing stations. These three weighing stations continuously measure the weight of a bowl, plate, and drinking cup or glass. Each weighing station consists of three triangle positioned measurement points (sensors) to balance weight. Besides these weighing stations, the mEETr tray includes a video-camera holder. Using a camera holder on the plate ensures that the camera is well positioned for the dietary and eating behavior measurements. Based on these video images, eating behavior characteristics can be determined as an extension of emotion detection software [53]. Eating behavior characteristics that can be determined are number of bites, sips, chews and swallows. Combining eating behavior information with weighing data of the meal facilitates calculation of bite size, eating duration per bite and eating rate, and the order in which different meal components are consumed. Tray weight and video data are transported to a PC using a wireless receiver. Here the data is cleaned after which outcome measures are calculated. The mEETr gives insight into how much people eat and the way food is consumed, which can be used for research purposes and to provide personalized eating behavior advice. More specifically, this could result in recommendations on food type (whole fruit instead of juices and smoothies) and eating rate, which adds to current dietary-assessment methods such as the 24hR, FFQ or food records that do not provide information on eating rate. Therefore, the mEETr is especially of use in specific population groups in which current dietary questionnaires cannot be used, such as in children [54,55].

### 4.2. Social Eating Behavior

Eating is not only about what, how much, and how food is consumed, it also about the social environment: ‘the social space of eating’. Sharing a meal can involve overt social aspects such as serving oneself or someone else food, passing on plates or serving trays, and adjusting overall dining time to table partners. There are also more subtle or covert social aspects involved such as going for seconds or thirds, synchronizing bites or eating speed, and synchronizing eating or serving quantity [56]. To understand the implicit social dynamics of eating together and how this impacts food choices, studies of social interaction are needed to also address social environmental factors in personalized dietary coaching. Social eating behavior is predominantly assessed through video recordings, which provides valuable insights into effects of conversations, eye contact between table members, or gesture mimicking [57]. However, video recordings miss out on dimensions like quantities of foods, which can relate to individual food intake but also food sharing. Although such dimensions can be monitored through weighing scales or instrumented cutlery, such attributes create awareness and may intervene with natural behaviors of the table members, and such introduce reactivity bias.

To address this, the University of Twente developed the Sensory Interactive Table (SIT) to measure both individual and social eating behaviors [58]. The SIT is an instrumented, interactive round dining table (⌀1.45 m). The table surface is composed of 199 individually controllable, hexagon-shaped modules, each embedded with a load cell (199 load cells total) and 42 LEDs each (8358 LEDs total) just below the tabletop surface. Each module is covered with hexagon-shaped 15 mm thick white plexiglass to diffuse the LED light. Modules are replaceable, providing the option to use other sensors and feedback modalities. A plastic foil is placed on top of the plexiglass to create a waterproof surface. A tablecloth makes up the last layer of the table to create a visually appealing unobtrusive measurement instrument [58]. The load cells measure the weight of the items on the table, over the course of a meal. As a result, many overt and covert aspects of eating behavior related to mass become measurable, such as bite size, total amount of food on a plate, or synchronicity of eating speed between individual table members. Additionally, the SIT provides the option for coaching through use of the LEDs, which allow for communication with table members by use of light interactions, potentially providing feedback and advice about their behavior, habits and eating choices [58].

Currently, the table is controlled through Unity (Unity Technologies), a cross-platform game engine that collects and processes the data from the loadcells (input) and control the interactions that is sent back towards the LEDs (output). The software allows for individual processing of input and output alone, or can interconnect the two, creating a feedback loop to the user. It creates a flexible set-up, suitable to study the eating behaviors of people in a social setting, the social interactions between people in a dining setting, and the continuous cycle of feedback and the response to this feedback in real time [58].

## 5. Technology to Improve Measures of Dietary Behavior

### 5.1. Image and Spectroscopy to Improve Dietary Intake Measures

In order to improve the quality of dietary intake assessment, a feature that may contribute to a more accurate dietary assessment is the use of images [59,60]. Images can be particularly interesting when tools are devoted to specific populations, such as children or individuals with intellectual impairment, as related to limited skills in terms of literacy, writing, food recognition and dependency of the care-giver [61,62,63]. Smartphone-based systems that estimate food intake by means of images already exist (e.g., [64]), but their usability still seems limited and results are often insufficiently validated.

Current efforts are particularly focused on two approaches, including image-assisted and/or food recognition image-based approaches. Image-assisted approaches are especially useful as part of retrospective methods (i.e., 24hR). Interviewees capture all food and drinks consumed through pictures, which subsequently assists the reporting of the foods and drinks consumed and associated portion sizes. However, for this approach image-review is required by the interviewee and/or researcher, which makes the method quite tedious. To circumvent this problem, automated food recognition (and volume) image-based approaches can be used as part of prospective methods (i.e., food records); interviewees record their intake by taking before and after pictures of all foods and drinks consumed. As image-based recording may facilitate automatic food identification and portion size estimation, this approach is especially promising to reduce respondent and researcher burden and increases the accuracy of current food and nutrient intake estimates by reducing reactivity bias, portion size errors and errors due to incorrect food identification. However, these methods still have many limitations as pictures must be taken from a specific angle and often in combination with a reference marker (i.e., to assess size and depth). Additionally, food recognition is still a challenge, especially in case of mixed-dishes or when differentiating between a diet soda and a sugar-containing version of that same soda [60,64]. Portion size estimation from pictures is also still a challenge as the weight estimate is based on food volume. However, volume is food specific, i.e., whereas a salad is voluminous and light, a candy bar is often dense and heavy [65,66]. Differences in food volume can be better assessed by using 3D-images over regular images. A 3D-camera, which is already part of the newest smartphone models, is able to detect the shape and volume of a food item. This allows the assessment of portion sizes.

Besides regular images, spectroscopic images of food products may serve future energy and macronutrient estimates of foods, so called chemical fingerprinting [67,68]. A spectroscopic camera can detect many different frequencies of light outside of the visible spectrum, which may facilitate product identification. Besides, spectroscopy uses near-infrared and infrared, which serve the assessment of food composition. The translation from chemical fingerprint to geometric and dynamic dietary information requires advanced ‘chemometric’ data-analyses techniques [59]. To determine a set of wavelengths needed to quantify macronutrient content, a 400–800 nm wavelength hyperspectral camera is needed. Such cameras with a limited wavelength range are expected to be incorporated in smartphones in the near future.

Thus, integrating a hyperspectral camera and a 3D-camera could provide more detailed food data, and lead to more objective measures of food intake.

### 5.2. Conversational Agents to Improve Dietary Intake Measures

Exploring the potential use of a conversational agent or chatbot could be another valuable supplementary input source to assess dietary intake, particularly among (older) adults with functional impairments (e.g., visual and/or motor impairment) and individuals with limited (E-)health literacy [69,70]. Implementation of a conversational agent may further simplify the recording process and increase accuracy [71,72].

A distinction can be made between rule-based chatbots and AI-based chatbots. Rule-based bots are the most common chatbots and are programmed according to a decision tree architecture; users have to answer specific (often closed) questions via text- and/or button-entry, after which the bot will respond based on the fixed decision tree. Therefore, rule-based bots are only useful for ‘simple’ tasks (e.g., tracking and stimulating fruit intake). In case of more complex tasks, AI-based chatbots are more suitable due to use of AI and Natural Language Processing (NLP), which enable more advanced ‘conversations’. By converting and interpreting text/speech and even images, AI-bots have the ability to make ‘intelligent decisions’, ‘learn’ (i.e., machine learning, deep learning) and provide better and more accurate answers in case of more long-term use [71]. Due to this learning process, AI-based bots are able to identify frequently consumed foods related to eating occasions and/or identify habitual consumption patterns, which in its turn enables the AI-bot to send personalized reminders at opportune moments to remember users to report their food intake.

Currently, multiple diet-related chatbots are available to assess and (often) influence dietary behavior [73,74,75,76], but these are not yet validated. Choi and Kim [77] evaluated the feasibility and acceptability of ICT based mobile chatbot technology to reduce dietary sugar intake: >60% of the participants reported difficulties related to the use of the chatbot and forgot to record their food intake, resulting in incomplete food intake registration. AI and machine learning techniques are assumed to further stimulate the use of conversational agents to assist dietary intake assessment, especially among specific target groups. Nevertheless, although chatbots may reduce participant burden, reporting an entire diet via chatbot may still be tedious due to the question-answer structure. Consequently, to further reduce participant burden, integrating the use of chatbots with other tools is needed. Utilization of available smartphone features and advanced AI and machine learning techniques are eminent to facilitate multiple data entry methods (text, speech, images), integrate personalized reminders and minimize reporting burden, but also to analyses the collected, often complex, data. These technological advances are not able to fully replace existing dietary assessment technologies but could be valuable additions to existing tools such as Traqq and have the potential to decrease reporting burden and improve accuracy, especially for specific target groups.

### 5.3. Video Image Analysis and Sensors to Improve Eating Behavior Measures

Whereas cameras provide opportunities to improve dietary intake assessment, advanced video image analysis techniques offer new opportunities in terms of automated detection of eating behaviors such as emotion detection (adults and children), acceptance and rejection behavior of infants and automated oral processing behaviors such as chews and bites in different age groups [78,79]. Deep learning models can be based on extracted data from the video, for example facial landmarks, and training the model on annotated events and their time. Alternatively, video images can be analyzed as a whole using all the available pixels as inputs to the model. This allows in context analysis, tracking all behaviors that occur when eating a family meal where interactions with the environment and all family members are taken into account [77]. The former may yield higher accuracy with simpler models and smaller datasets, and the latter may allow more comprehensive machine learning of the full complexity of eating behavior. Besides video processing, eating behavior can also be assessed using newly-developed sensors such as a wristband with an IMU sensor to detect the hand-movement bringing food to the mouth [80,81]. In the future, eating events may also be detected using other sensor wearables such as headbands like Muse^TM^, which measures EEG, PPG heartrate and accelerometry. Based on input from all these different sensors eating episodes or chewing may be detected using prediction analysis such as a neural networks (AI).

## 6. Technology to Improve Personalized Dietary Behavior Interventions

### 6.1. Conversational Agents for Stimulating Dietary Behavior Change

In addition to using conversational agents for dietary assessment, conversational agents are also used to stimulate dietary behavior change. However, considering existing apps using this technology, it can be concluded that the level of personalized advice is limited. Users are often referred to health professionals for more detailed advice [71]. Integrating AI-based chatbots will enable the detection and visualization of dietary patterns, both graphical and textual, as well as the provision of real-time personalized suggestions and goal setting to promote small daily changes. Such changes or goals could apply to changes in dietary intake as well as eating behavior. More specifically, in terms of eating behavior, the chatbot could pop questions related to appetite and feelings of hunger, which could translate into portion sizes suggestions, or a timer could be initiated to motivate the user to eat more slowly.

Additionally, embodied conversational agents (ECAs) also seem promising in stimulating and maintaining health behavior change [82]. ECAs are animated computer characters (i.e., avatars) that are able to establish and maintain a more personal relationship with the user [83]. Research indicates that face-to-face coaching seems to be more effective in establishing long lasting behavior change [19]. As ECAs can mimic face-to-face coaching and are available 24/7, ECAs can offer coaching when it is needed most. Moreover, motivational features can stimulate the user to adhere to the recommendations and, for instance, support users with shopping in a healthy and sustainable way [84]. Additionally, avatars can also be used to communicate feelings of hunger and satiation to teach children, for instance, when to stop and start eating through imitation, establishing healthy eating behavior from an early age [85]. Therefore, implementing an AI-based chatbot or ECA into existing tools such as Traqq can be very valuable in enabling tailored diet coaching [82,83]. Future research should address how the use of ECAs can be optimized in order to create actual behavior change. Regular interaction is a prerequisite, but like other eHealth tools, uptake is limited. Studies show that appearance of the ECA matters, but even when appearance and design is optimized, the dialogues need to remain persuasive and engaging over time. It might well be that conversational agents for dietary change could function best in an add-on format to other interventions (e.g., in addition to regular care or dietician’s advice) or for specific patients group that are highly motivated to adjust their diets (e.g., patients in cardiac rehabilitation) [86,87,88].

### 6.2. Game-Elements to Improve Dietary Behavior Interventions

Another promising and attractive strategy to increase intrinsic motivation and engagement of diet-related tools is via games (i.e., serious games) or game elements (i.e., gamification) [89,90]. By harnessing the ‘fun factor’ of games, enjoyment in using diet-related tools can be increased and in such way users’ motivation and engagement can be fostered. Moreover, digital tools can become easier to use and better understandable [90,91]. Although research on effectiveness is still in its infancy, reviews show promising results of gamified interventions to promote healthy behaviors and, specifically, the promotion of a healthy diet, both in children and in adults [92,93,94]. An example of a gamified intervention is the serious game ‘Squire’s Quest!’ that encourages children to consume more fruits and vegetables. The purpose of the game is to go from squire to knighthood and for this the squire (i.e., child) had to overcome multiple challenges. These challenges consisted of attaining real-word fruit and vegetable consumption-related goals. By successfully completing these challenges, the child earned badges and progressed towards knighthood [95]. Squire’s Guest! contains a variety of integrated behavior change techniques to promote self-efficacy and intrinsic motivations (e.g., goal setting, planning, self-monitoring, goal review and feedback), which are key mediators of behavior change in children [96]. To optimize chances of app engagement and effectiveness it is important to match the game or gamified tool to the user’s needs and preferences, therefore, it is recommended to follow a user-centered design approach when developing gamified diet-related tools [97,98].

### 6.3. Targeted Dietary Interventions during Food Shopping

Trustworthy diet-related apps also have the potential to deliver real-time dietary advice to users when food shopping. For example, users receive personal dietary advice, e.g., specific product recommendations, recipes, grocery lists, tailored to the assortment while shopping in an online grocery store or when using a smartphone or handheld scanner devices in a physical store.

To date, nutrition health apps (i.e., apps aiming at improving users’ health) and nutrition information apps (i.e., apps aiming at delivering transparent nutrition information of food products) [99] exist that help make smarter food purchases. These have mostly been developed by researchers [100,101], governmental organizations [102], retailers, or other organizations. Most of these dietary recommendations apps focus on tracking weight loss, or specific health conditions over a longer period and aim to educate users to adopt a healthier lifestyle. However, few focus on the specific context of food consumption and purchasing moments [99,101,103,104,105,106]. In addition, most apps do not include personalized dietary advice but are based on general nutrition guidelines.

It is assumed that app-based interventions that do not solely focus on educating but include more concrete coaching in the form of eating tips, recipes, and grocery lists (e.g., for specific food retailers) make it easier to adopt behavioral change [105]. A practical and tailored grocery shopping list can assist in healthful shopping. An example, ‘MyNutriCart’, is a smartphone app that helps users select healthier foods based on the U.S. Dietary Guidelines and on their budget [100,106]. It offers users a practical grocery list fitting the dietary nutritional recommendations and taking into account the caloric requirements based on their weight goal (i.e., loose, gain or maintain weight) [100] and led to improvements in terms of healthy food-related behavior [106].

Integrating such approach into a dietary assessment app like Traqq could also simplify the reporting of food intake. Recommended recipes and grocery lists can be stored in the app and consulted during food intake reporting. Consumed recipes or products can then easily be transferred to the food record or recall module of the app.

### 6.4. Tailored Dietary Behavior Interventions

While most food intake recommendations follow a one-size-fits-all approach catered to a population or subpopulation, personalized dietary advice provides recommendations tailored to the individual. Therefore, personalized dietary advice fits better with individual needs and leads to a more effective approach towards long-term change in dietary behaviors [107,108]. In order to personalize advice, various individual characteristics and their interactions can be taken into account such as educational level, social and economic status, current nutritional status (i.e., nutrient deficits and surpluses), and individual lifestyle behaviors and preferences. Provision of personalized advice may offer opportunities for tailored interventions with superior health benefits aligned to the individual’s nutritional status and a better compliance with the advice through better alignment with individual lifestyles and preferences. Examples of such a personalized intervention are Just-In-Time-Adaptive Interventions (JITAIs). JITAIs adapt their support “overtime to an individual’s changing status and contexts”, aiming to deliver support “at the moment and in the context that the person needs it most and is most likely to be receptive” [109]. Data from smartphones or wearables are used to automatically and continuously acquire information about the user and its context (e.g., environmental exposures) and deliver individualized interventions based on states of vulnerability and receptivity of the user [110,111]. Based on these data the delivery of the intervention elements can be continuously tailored towards the specific status and context of the user [112]).

Additionally, to provide tailored dietary interventions, it is crucial to adopt behavior change theory to identify and influence key constructs related to behavior change. Currently, the integration of behavior change techniques (BCTs) in diet-related apps is often lacking [24,25,26]. Although diet-related apps vary greatly in the number of integrated BCTs, goal setting, self-monitoring and feedback are integrated most frequently [24]. These BCTs have been proven effective in general weight loss interventions [113,114,115]. However, these techniques all relate to behavioral control and do not focus on other constructs such as development of essential behavioral skills [24]. More recently, Villinger et al. [116] reviewed the effectiveness of app-based diet-related behavioral interventions and concluded that additional intervention components besides the app and a higher number of implemented BCTs did not necessarily improve the effectiveness of the interventions. Thus, the effectiveness of an app-based diet intervention will not be determined by the quantity of implemented BCTs, but by their quality. The design and the technical implementation of a BCT can influence effectiveness of an app [117]. Implementing a variety of BCTs will enable the user to tailor the app to their preferences and develop a personalized intervention.

## 7. Discussion

This paper discusses technological opportunities to improve dietary behavior assessment and interventions. The P&P consortium offers a unique combination of disciplines, which is needed to improve dietary behavior assessment and subsequently tailor interventions to establish lasting dietary behavior change. P&P’s current efforts have led to the development of tools such as Traqq, mEETr and SIT. These tools allow for assessment of dietary intake, eating behavior and social or contextual dietary behaviors.

Developing targeted dietary behavior measures and connecting these to behavior change interventions is key to the establishment of lasting behavior change in order to ultimately improve health. Specific target (sub)populations have explicit (nutritional) requirements, and behavior change efforts should be tailored to the individual. Consequently, various individual characteristics should be taken into account such as age, culture, social-economic status, personality trait and level according to the theory of behavior change [118]. During the development of tailored interventions or tools it is imperative to take these requirements in to account. Currently, P&P efforts are focused on four main target populations.

The first target group of the P&P consortium are pregnant, lactating women and their (unborn) children. Although a healthy diet during pregnancy and lactation is important to ensure optimal supply of various nutritional sources, the diet of pregnant and lactating women is often suboptimal [116,119,120], which stresses the need for more effective personalized approaches. The new technologies as described in this paper offer the opportunity to develop more personalized approaches for pregnant and lactating women. Another window of opportunity to stimulate a healthy diet is childhood, which highlights the second target group of the P&P consortium, namely children. Currently, dietary assessment among young children is challenged by limited reading and writing skills as well as food knowledge of the children themselves. As a result, healthcare professionals and researchers depend on the caregivers. Therefore, P&P aims to develop practical technological tools to optimize dietary intake assessment and guidance of (future) mothers and their young children. The third target group of the P&P consortium are older adults, who have specific nutritional needs, often limited digital capabilities, and prefer the more personal approach [75]. Human-computer interaction design seems promising here, e.g., using ECAs that are able to mimic face-to-face coaching. A fourth target group of the P&P consortium are office workers, who spend a large proportion of their time at work where they consume about a third of their daily energy intake [121,122]. The office environment is ideal to set up dietary interventions due to fixed work schedules and a generally limited access to food items and meals [121,123].

To arrive at personalized diet-related tools, P&P not only focuses on end-users but also on stakeholders (e.g., health and nutrition professionals, commercial firms, government bodies). We investigate underlying values that play a role for different stakeholders; uncovering values provides insight into how people wish to live their lives and what matters most to them. Stakeholder values and any potential tensions between co-existing values need to be taken into account in the design of interventions. In part, the focus needs to be on the worth that is created for different parties [124]. For example, for users there could be worth in having to spend less time on their diet, while for health and nutrition professionals a better insight into the nutritional needs of different target groups could be of interest. However, stakeholders’ internal values also need to be taken into account, such as the need for privacy, or being able to autonomously decide about food intake [124]. We feel that adopting a multi-stakeholder, value-based approach is crucial to arrive at tools that can be seamlessly integrated into people’s daily lives for long-term use, and that will be supported by the ecosystem of stakeholders involved.

To achieve personalized technology-assisted dietary interventions, supporting technologies need access to relevant data on the individual. This implies that individuals have to be willing to engage in an information exchange process with the diet-coaching app [125]. People have to share personal, often sensitive information with the app, which induces (perceived) privacy risks for users in order to receive tailored advice. Therefore, the use of user-documented food consumption data also raises specific legal and ethical challenges [22]. The trade-off between the perceived personal benefit of this advice and the perceived privacy risk of sharing personal information will affect whether or not users adopt the diet-coaching app [125,126]. Therefore in developing apps to facilitate personalized advice, characteristics such as the type, amount and sensitivity of personal information disclosed, advice scope (e.g., personal diet plan, personalized shopping list, religious taboos, personal allergies), the trustworthiness of the communication channel, business model, and service provider need are to be taken into account [125,127].

Nowadays, technology advances rapidly which results in new technological opportunities to improve dietary behavior assessment and intervention. However, it also brings along new challenges. Due to the rapid technological evolution, it is difficult to stay up-to-date. New quickly becomes outdated. To ensure the quality of the tools we develop, we focus on techniques that have been proven effective in either dietary assessment or dietary behavior change. We do not attempt to develop new techniques but implement existing techniques. To further ensure the accuracy and effectiveness of the tools, thorough evaluation is an important aspect of the developmental process.

## 8. Conclusions

Technological innovations offer the opportunity to improve dietary behavior assessment and interventions. Moreover, they enable targeted dietary behavior interventions, tailored to individuals, specific target groups or situations (e.g., during food shopping). Advanced image and video processing in combination with AI and machine learning techniques will be explored to improve current dietary behavior measures and to reduce registration burden and improve accuracy. Integration of conversational agents (i.e., chatbots, avatars) and game elements in existing systems such as Traqq show promise in tailoring dietary behavior coaching to improve engagement. Finally, targeted dietary behavior interventions can be improved by integrating behavior change techniques and tailoring to the individual, target group, or situation. Utilization of these technological innovations in dietary behavior assessment and interventions has the potential to significantly improve the healthiness of individuals’ eating behaviors.

## Data Availability

Not applicable.
